# Renal Parenchymal Damage and Persistent Hematuria after D-J Insertion: A Report on Two Cases

**DOI:** 10.2174/0115734056338723241202053717

**Published:** 2025-04-17

**Authors:** Muhammed Cihan Temel

**Affiliations:** 1 Department of Urology, Nevsehir State Hospital, Nevsehir, Turkey

**Keywords:** Double-J stent insertion, Renal parenchymal perforation, Malposition, Case report, Kidney Urine Bladder, Nonsteroidal anti-inflammatory (NSAI) therapy

## Abstract

**Introduction/Background:**

In this case series, we present two male cases with renal parenchymal perforation without perirenal hematoma after D-J ureteral stent insertion, one with nutcracker renal vein syndrome. Our study provides new and important contributions to the field of science regarding what to consider during D-J stent insertion in similar cases and in patients with obstruction in the urinary collecting system for more than 2 months.

**Case Presentations:**

Two patients, 30 and 37 years old, who were inserted a D-J catheter after endoscopic ureteral stone treatment, suffered from severe ipsilateral flank pain and hematuria after the operation. The Kidney Urine Bladder (KUB) radiography showed that the proximal part of the D-J stent was protruding from the upper calyx and parenchyma of the kidney in both patients. One of the patients had an ipsilateral nutcracker renal vein syndrome, and the clinical progression was more severe. In both cases, conventional follow-up with bed rest, nonsteroidal anti-inflammatory (NSAI) therapy, intravenous (IV) fluid infusion, and anti-biotherapy after the D-J stent reposition was sufficient. The patients had no clinical problems during the next outpatient clinic visits.

**Conclusion:**

Double-j (D-J) ureteral stent insertion procedure may cause many life-threatening complications, from subcapsular hematoma to pulmonary embolism. In this case series, conventional follow-up was sufficient for the treatment of patients with renal parenchymal damage without perirenal hematoma due to D-J stent insertion, including nutcracker renal vein syndrome cases. More care should be taken when placing D-J stents, especially in patients with obstruction in the urinary collecting system for more than 2 months and with nutcracker renal vein syndrome. In these patients, the soft proximal end of the guidewire should not be pushed and forced too hard to the upper part of the kidney and upper collecting system. Additionally, the D-J stent placement procedure should be performed under fluoroscopy as much as possible to avoid complications.

## 1 INTRODUCTION

In today's practice, Double-J (D-J) stent insertion is a commonly used procedure after endourological surgery. D-J stent insertion is frequently performed in cases of gynaecological tumour, trauma, retroperitoneal fibrosis, renal transplantation, pregnancy hydronephrosis, SWL treatment, and after ureteroscopy operations [1]. However, like any invasive procedure, D-J insertion also has complications [2]. Even in ureterorenoscopy (URS) and stent placement, which are performed very often and are very straightforward, life-threatening complications might occur [3, 4]. In this case series, we present two male cases with renal parenchymal perforation without perirenal hematoma after D-J ureteral stent insertion, one with nutcracker renal vein syndrome. These rare complications seem minor but may increase patient and surgical stress significantly and might be life-threatening. Our study provides new and important contributions to the field of science regarding what to consider during D-J stent insertion in similar cases and patients with obstruction in the urinary collecting system for more than 2 months. It also confirms and supports the rule that “D-J stent placement procedure should be performed under fluoroscopy to avoid complications as much as possible” [5, 6].

## 2 CASE REPORTS

In the last year, persistent hematuria developed in two different cases in which a D-J stent was inserted due to ureteral stone treatment in our center. Both patients had pre-operative hydronephrosis, and the operations were performed by a single urologist who is experienced in the field of endourology. Stones of both patients were causing obstructions and hydronephrosis for more than 2 months. There was no bacterial growth in the urine cultures taken during the preoperative period, and the D-J stent insertion was performed without using fluoroscopy. Helsinki Declaration has been followed for involving human subjects in this case series.

### 2.1 Patient 1

**Fig. (1):**
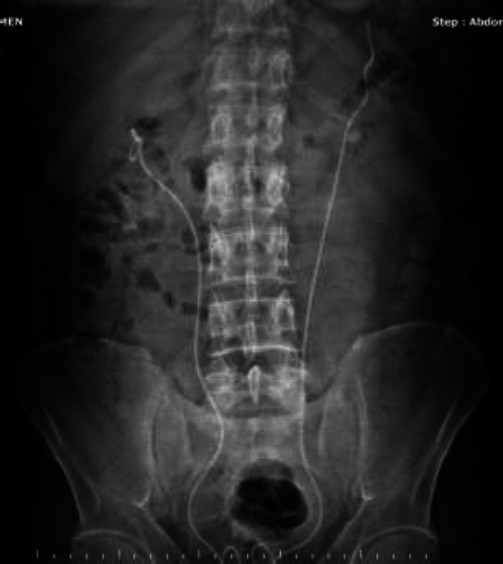
KUB radiography of Patient 1 taken on the 1st postoperative day.

A 37-year-old male patient underwent right endoscopic ureter stone treatment and bilateral D-J stent insertion using a semirigid ureterorenoscope due to 8 and 9 mm calculi at the right ureterovesical junction, a 12.6 mm calculus at the left ureteropelvic junction, and bilateral grade 2 hydronephrosis. The standard bilateral endoscopic ureterolithotripsy operations lasted 40 minutes and were completed without any problems. Bilateral 4.8 Fr, 28 cm D-J stents were inserted into both ureters over the 0.035-inch hydrophilic sensor guidewires without any problem. We do not use fluoroscopy and contrast images standardly in every case due to their radiation effects and handling difficulties in our clinic. Therefore, for this patient as well, we did not use fluoroscopy and contrast imaging as we did not find it necessary. The patient had no additional disease or comorbidity except for Hepatitis B. Hepatitis B disease was not in the active phase, and the patient was not receiving any treatment. The patient had bilateral flank pain (more severe on the left side) and hematuria on the 1^st^ postoperative day, and the kidney urinary bladder (KUB) radiography showed that the proximal part of the left D-J stent was protruding from the upper calyx and renal parenchyma (Fig. **1**).

**Fig. (2):**
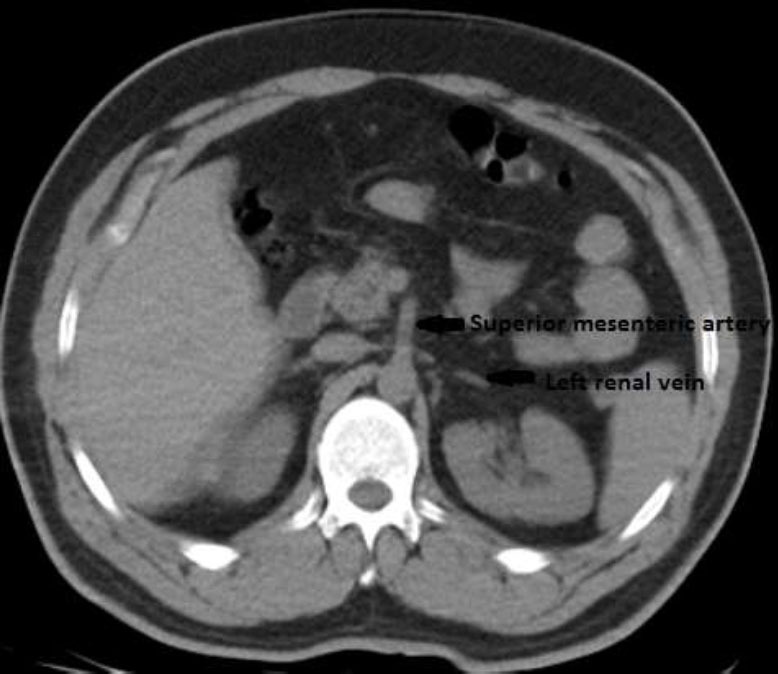
Nutcracker renal vein syndrome in abdomen CT.

On the postoperative 1^st^ day, the left D-J stent was slightly withdrawn under local anesthesia to place the proximal end of the left D-J stent into the collecting system. However, when it was observed that the proximal end did not create a loop view, the stent was thought to be outside of the renal parenchyma and was removed completely. After these procedures, the hematuria and the patient's bilateral flank pain continued until the postoperative 3^rd^ day, and it tended to increase, especially after mobilization. Severe hematuria was observed in the patient on the 4^th^ postoperative day, and triphasic computed tomography (CT) angiography was performed after bladder irrigation. CT angiography showed that the left renal vein was localized under the superior mesenteric artery (nutcracker left renal vein syndrome) (Fig. **2**).

It was thought that persistent hematuria and flank pain were due to damage of the renal parenchyma, which was due to high blood pressure due to nutcracker left renal vein syndrome, and the patient was followed up with bed rest, NSAI treatment, intravenous hydration, and anti-biotherapy (Cefuroxime axetil 2 x 500 mg IV). While the patient's preoperative hemoglobin was 18.3 g / dL, his hemoglobin on the postoperative 5^th^ day was 14.9 g / dL, and there was no left flank pain or need for blood transfusion during follow-up. However, on the postoperative 5^th^ day, the patient, whose mild hematuria and right flank pain persisted, underwent cystoscopy again under spinal anesthesia. During cystoscopy, hyperemic and bleeding areas were seen in the bladder neck, and bipolar electro-fulguration was performed on these areas. No bloody efflux was observed in the bilateral ureteral orifices. However, because the patient's right flank pain continued, the right D-J stent was also extracted, and the right diagnostic URS was performed. No additional pathology or bleeding focus was observed along the right ureter except for small calcular fragments and tissue debris. A 22 Fr 3-way urethral catheter was inserted into the patient, and the operation was terminated. The patient could adhere to all interventions and treatments. When a more detailed history was taken from the patient after the operations, he stated that he had occasional hematuria complaints also before operations and that he attributed it to renal stones. In addition, it was also learned that he could not tolerate the D-J stent due to pain in his previous URS operations. The patient, whose hematuria and side pain regressed after cystoscopy and withdrawal of the right D-J stent, was discharged on the 7^th^ postoperative day. The patient did not have any clinical problems during the next outpatient clinic visits.

### 2.2 Pati̇ent 2

**Fig. (3):**
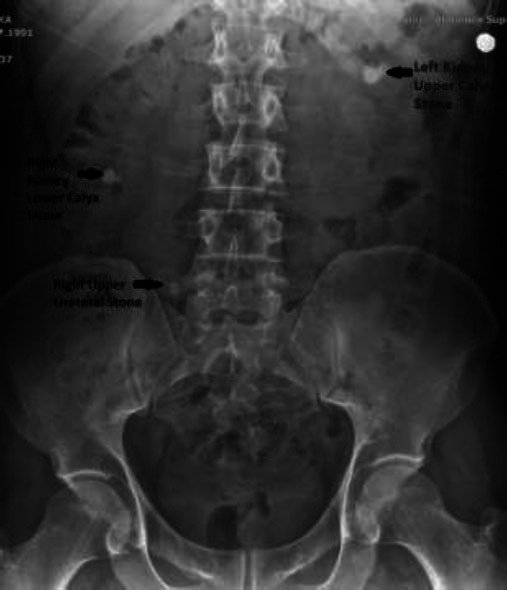
Preoperative KUB radiography of patient 2.

**Fig. (4):**
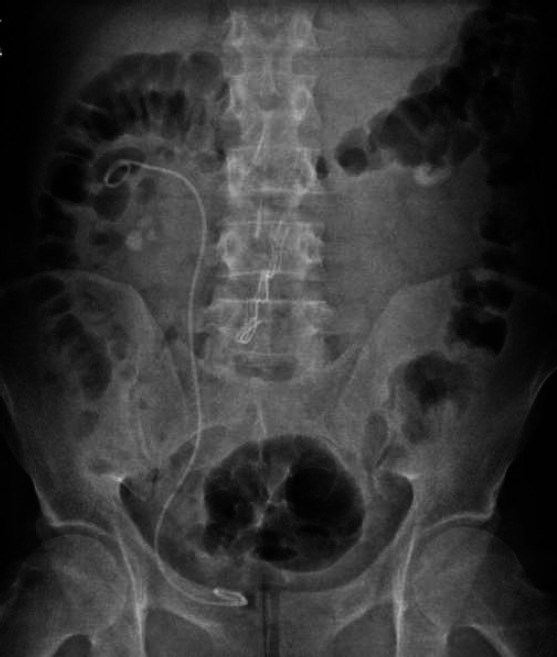
KUB radiography taken on the same day after the operation.

A URS operation was performed on a 30-year-old male patient for a 9.3 mm calculus in the right upper ureter and right grade 2 hydronephrosis (Fig. **3**). As the stone was pushed up during the operation, laser lithotripsy could not be performed, and 4.8 Fr, 28 cm D-J stent insertion over the 0.035-inch hydrophilic sensor guidewire was performed. D-J stent insertion was completed without any problems, and the operation lasted 20 minutes totally. We also did not use fluoroscopy and contrast imaging in this patient. The patient had no additional disease or comorbidity. However, the patient developed severe right flank pain and hematuria on the evening of the operation. A KUB radiography was taken on the same day, and it was thought that the right D-J stent had rotated in the upper calyx and it might have caused calyceal damage during stent placement and the patient was followed up with bed rest, NSAI treatment, and intravenous hydration (Fig. **4**).

Since there is no ethical approval requirement for case reports in our country, we did not obtain ethics committee approval for this manuscript. Helsinki Declaration was followed to involve human subjects in this study.

**Fig. (5):**
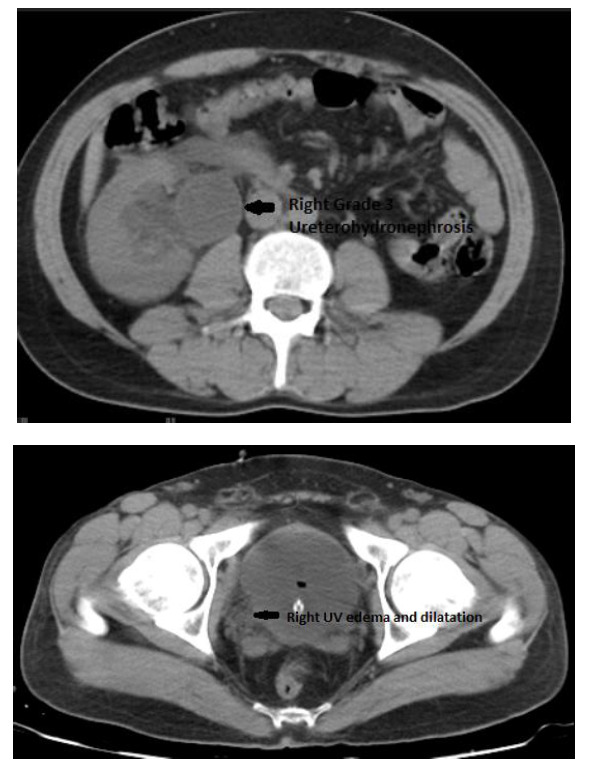
Abdomen CT image on the 2nd postoperative day, after D-J is withdrawn.

The patient's flank pain and hematuria became more severe on the postoperative 1^st^ day. Although IV Dexketoprofen 2 x 50 mg and IM Pethidine Hydrochloride 2 x 50 mg were administered to the patient, the flank pain did not completely regress. So, the right D-J stent extraction was performed under local anesthesia. After these procedures, the patient's flank pain and hematuria regressed. On the postoperative 2^nd^ day, the patient's hematuria resolved itself completely, but an increase in right flank pain was observed again. Thereupon, an abdominal CT scan was performed on the patient. Computed tomography showed grade 3 hydroureteronephrosis and edema in the ureterovesical (UV) junction at the right side. Antero-posterior (AP) diameter of the right renal pelvis was measured as 35 mm (Fig. **5**).

**Fig. (6):**
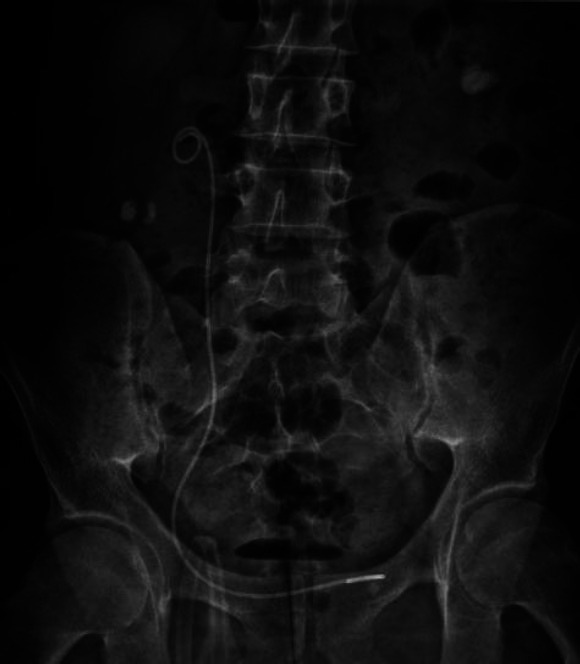
KUB radiography of Patient 2 on the postoperative 3rd day, after Re-Double-J stent insertion.

Due to the right ureterohydronephrosis and the persistent right flank pain, right diagnostic URS was performed, and a right D-J stent was inserted again on the 2^nd^ postoperative day. We did not use a different technique for the second D-J placement than we did for the first D-J placement. We used the same type of hydrophilic sensor guidewire for both insertions. However, unlike the first placement, we used real-time fluoroscopy for the second D-J stent placement, and we finalized the procedure after ensuring that the proximal loop of the D-J stent rotated in the right renal pelvis. Additionally, in this second D-J insertion, we did not push or force the soft proximal end of the guidewire to the upper part of the kidney and upper collecting system. No additional pathology was observed except for right UV junction edema in URS. After this procedure, flank pain and hematuria were significantly reduced in the patient. The patient could adhere to and tolerate all interventions and treatments. The patient was discharged on the postoperative 3^rd^ day after the KUB radiography showed that the D-J stent was in the right place (Fig. **6**). The patient had no clinical problems in the next outpatient clinic visits.

## 3 DISCUSSION

In current urology practice, D-J stent placement is a widely used standard procedure after endourological interventions. Moreover, D-J stent insertion is frequently performed in cases of gynecological tumor, trauma, retroperitoneal fibrosis, renal transplantation, pregnancy hydronephrosis, and SWL treatment [1]. However, it can lead to severe complications, such as dislocation, migration, calcification, encrustation, subcapsular hematoma, and infection [6-9]. Geavlete P. *et al*. stated in a comprehensive study that 0.3% of common complications of D-J stent insertions are D-J stent malpositions. They also stated that proximal D-J migration was seen in 0.9% of cases, and distal D-J migrations were seen in 0.7% of all cases. The obstruction of the ureteral stent, causing inefficient drainage, was encountered in 1.85% of cases, while irritative bladder symptoms occurred in 32.7% of cases. Hematuria was observed in 10.38% of cases. Urinary tract infection was diagnosed in 14.8% of cases. Stent encrustation and calcification were occurred in 1.66% of cases [10].

In this study, we aimed to present two different cases that have persistent hematuria and renal parenchymal damage due to D-J stent placement after endoscopic ureter stone treatment. The cases of renal parenchymal perforation due to D-J insertion without perirenal hematoma are very limited in previous literature [11, 12]. In both cases we presented, renal parenchymal damage due to D-J stent was observed without perirenal hematoma or urinoma. In addition, in our 1^st^ case, left flank pain and hematuria were more severe due to the presence of the left nutcracker renal vein syndrome, and this case is a first in the literature in terms of this feature. Renal parenchyma or calyx damage complications must be kept in mind where there is unreasonable flank pain and persistent hematuria after D-J stent insertion in endourological procedures. In addition, it is a misconception that almost all urologists think that the D-J stent is in the right place when they examine that the proximal end of the D-J stent becomes a loop shape on fluoroscopy or KUB radiography. As in our 2^nd^ case, the proximal end that looped may have rotated inside a ruptured/damaged calyceal system or outside the collecting system.

In these cases, we have seen how the insertion of a D-J stent, which seems like a simple procedure, can lead to serious complications, such as severe hemoglobin decrease, severe flank pain, and severe hematuria. Early detection of such complications will prevent the case from becoming even more complicated. The presence of pain, fever, or gross hematuria in the postoperative period should arouse suspicions in the surgeon. Normally, fluoroscopy is recommended during D-J stent insertion [5, 6]. However, we perform too many endoscopic operations and insert too many D-J stents in our daily clinical practice. So, we do not use fluoroscopy in every case due to its radiation effects and handling difficulties. Routinely, we check whether the D-J stent is in the right place with KUB radiography on the first postoperative day. If we suspect any problems during D-J stent insertion during operation, we check with fluoroscopy at the same time. However, based on the findings in our cases, it can be said that it is substantially more appropriate to do this procedure under fluoroscopy during the surgery, wherever possible. We recommend that surgeons perform all D-J stent insertion procedures under fluoroscopy in the following process. In centers where there is no fluoroscopy, postoperative KUB radiographs should be used definitely to check that the stent is in the right position. In cases where the stent is suspected to be in the wrong localization, cross-sectional studies should be made with CT to determine the location of the stent. As seen in our second case, although the proximal end of the D-J stent was found to be looped in the KUB radiogram, it was actually located outside the collecting system in CT images.

In impacted ureteral stones that cause obstruction for more than two months, the resulting inflammation increases the fragility of the renal parenchyma, pelvis, and calyces. The risk of renal perforation increases depending on this situation. The upper urinary tract obstruction caused by urinary calculus has led to increased renal pressure and vulnerability of the renal parenchyma, increasing the risk of parenchymal renal rupture. Additionally, it is thought that subclinical infections caused by obstruction may also make the kidneys fragile [13-15]. In our two cases, ureteral stones were causing obstruction for more than 2 months. We think that this situation contributes to the damage to the renal parenchyma and the collecting system. Additionally, we think that in both patients, we may have pushed and forced the soft end of the guidewire toward the kidney too much. We may have done this instinctively to minimize the possibility of the proximal end of the D-J stent looping in the upper ureter. We believe that this incorrect application also contributed to parenchymal and collecting system damage. It can be concluded that also it is highly likely that the nutcracker syndrome in the first patient contributed to this damage.

The limitations of this article were that it was planned as a case series, contrast images were not taken from patients during D-J stent placement, and fluoroscopy images were not taken immediately after D-J insertion.

In addition to such limitations, it has some strengths. Our study is the first in the literature to present these two rare cases. In summary, in both of our cases, the soft tip of the guide wire damaged the renal parenchyma for a reason that we cannot fully determine. Our study may not have provided clear and definitive statistical results, but it has shed light on potential scientific research that can be conducted in the future. It also provided at least some concrete evidence on how surgeons should act in cases like these. We believe that more comprehensive and randomized studies should be conducted in the future to further embody the results of our study. In addition, studies conducted on D-J stent insertion complications can be reviewed to obtain more precise and clear information on the mechanisms and prevention of these complications. Moreover, animal experiments can artificially create the factors that cause these complications and provide more valuable and clear information to the field of science.

## CONCLUSION

D-J stent insertion, which is frequently applied after endourological surgeries, may rarely cause life-threatening complications from pulmonary embolism to perirenal hematomas requiring transfusion. In cases of persistent flank pain and hematuria after D-J insertion, renal parenchyma or calyx damage should definitely be kept in mind, and when detected, following up with D-J stent reposition, bed rest, NSAI therapy, IV hydration, and antibiotherapy should be started at the first stage. As reported in the previous literature and our cases, these treatments are sufficient in most of the cases. More care is necessary when placing D-J stents, especially in patients with nutcracker renal vein syndrome and in cases that have collecting system obstruction for more than two months. In these patients, the soft proximal end of the guidewire should not be pushed and forced too hard to the upper part of the kidney and upper collecting system. In order to realize the results of our study further, more comprehensive, systematic, complex studies and even animal experiments are needed.

## PATIENTS’ PERSPECTIVE

**Patient 1: **I went through a difficult and challenging process due to the treatment of my stones and was discharged home after my doctor's long efforts. I thank everyone who contributed to my treatment.

**Patient 2:** I thank my doctor for doing everything he could for my treatment.

## Data Availability

All data generated or analyzed during this study are included in this published article.

## LIST OF ABBREVIATIONS

D-J: Double J
KUB: Kidney urine bladder
NSAI: Nonsteroidal anti-inflammatory
IV: Intravenous
URS: Ureterorenoscopy
CT: Computed tomography
Fr: French
IM: Intramuscular
UV: Ureterovesical
AP: Antero-posterior
SWL: Shock wave lithotripsy

## STANDARDS OF REPORTING

CARE guidelines were followed.
